# Fat necrosis associated with the use of oral anticoagulant therapy: atypical mammographic findings

**DOI:** 10.1590/0100-3984.2015.0007

**Published:** 2016

**Authors:** Ricardo Schwingel, Orlando Almeida, Tiago dos Santos Ferreira

**Affiliations:** 1 Faculdade de Ciências Médicas da Universidade Estadual de Campinas (FCM-Unicamp), Campinas, SP, Brazil.

Dear Editor

We report the case of a 54-year-old female with systemic lupus erythematosus, lupus nephritis, antiphospholipid syndrome, and deep vein thrombosis, who was being treated with an oral anticoagulant and a corticosteroid, as well as receiving immunosuppressive therapy. Her international normalized ratio was between 2 and 3, and she presented with recurrent spontaneous hematomas. She had been diagnosed 20 months prior with miliary pulmonary tuberculosis, which had been treated for 12 months. After the patient had undergone mammography (Figure 1), we reviewed the clinical data: she reported a recent spontaneous left-sided hematoma, with palpable nodules and ecchymosis, in the superolateral quadrant. As can be seen in Figure 2, ultrasound with Doppler flow imaging showed correspondence between this findings and an irregular hypoechoic nodule with indistinct margins without vascularization, measuring 6.0 × 3.0 cm, associated with architectural distortion, in the superolateral quadrant-together with images suggestive of lipid cysts. Initially undetermined, the lesion was considered likely benign, suggestive of fat necrosis, probably associated with anticoagulant use and hematoma formation. To avoid biopsy, we opted for a strategy of observation only.

**Figure 1:**
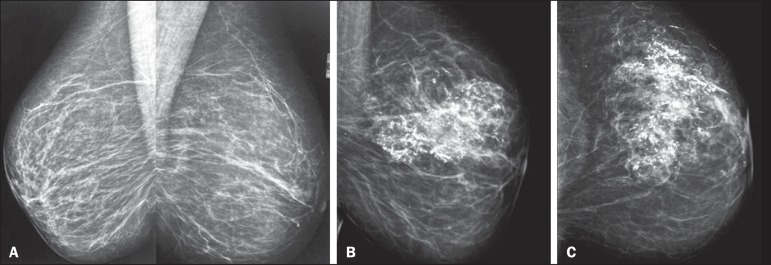
**A.** Screening mammography, from two years prior, in right and left mediolateral oblique views, showing sparse, bilateral, punctate vascular calcifications (BIRADS 2). **B,C:** Current mammograms of the left breast, in mediolateral oblique and craniocaudal views, showing global asymmetry in the left breast (the previous findings persisting in the right breast), accompanied by coarse dystrophic calcifications in the superolateral quadrant-an atypical pattern in fat necrosis.

**Figure 2:**
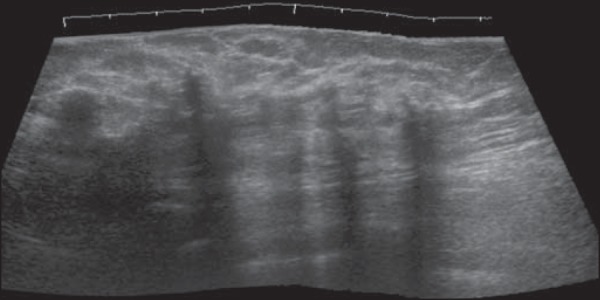
Ultrasound of the left breast, showing an area of architectural distortion accompanied by irregular hypoechoic nodule with indistinct margins, which attenuates the ultrasound waves, without detectable vascularization on the Doppler flow study, together with adjacent round, circumscribed nodules, with posterior acoustic shadowing, giving the appearance of lipid cysts.

Fat necrosis is often silent, appearing only as an abnormal mammographic finding. In rare cases, it can manifest as a palpable mass without associated mammographic findings. It is typically secondary to incidental or iatrogenic trauma and can occur in patients who are using anticoagulants or even in those without a relevant history.

Mammography is the most important test in the assessment of fat necrosis. Depending on the stage and the amount of fibrosis, it can manifest as a lipid cyst or as features that simulate malignancy: spiculated hyperdense areas; nodules accompanied by skin retraction; coarse calcifications; or clusters of microcalcifications. In the case presented here, the dominant findings were calcifications/microcalcifications and global asymmetry^(1-3)^.

On ultrasound, fat necrosis has several presentations, some more suggestive of benignity, such as subcutaneous hyperechogenicity (mainly associated with trauma) or an echogenic band within a lipid cyst with a position-dependent orientation. When fat necrosis manifests as a hyperechoic nodule, other features suggestive of benignity or malignancy should be examined, such features including orientation (parallel or perpendicular), shape, margins, posterior acoustic shadowing (absence or presence), and compartment^(2,3)^. In the case presented here, the presentation as a hypoechoic nodule permeated with lipid cysts supports benignity, although the indistinct margins could raise the suspicion of malignancy. In cases of fat necrosis, the most common evolution is the coalescence of coarse calcifications and normalization of subcutaneous hyperechogenicity, together with the development of anechoic areas and the transformation of lesions from complex to cystic. However, the lesions can either grow or stay solid. In inconclusive cases, needle aspiration of lipid material can facilitate the diagnosis, biopsy being reserved for use in cases in which the aspiration yields bloody fluid^(1-3)^.

Lupus mastitis, which occurs in 2% of patients with systemic lupus erythematosus, can be considered in the differential diagnosis of fat necrosis. It can present as palpable nodules accompanied by progressively larger and coarser calcifications on mammography, reflecting the evolution from focal panniculitis to fat necrosis, occasionally accompanied by axillary lymphadenopathy^(4-6)^.

Although mammary tuberculosis would be another possible diagnosis, the patient reported no pain, which is common in mammary tuberculosis. In the case presented here, mammography did not reveal a coarse stromal texture (the most common pattern), signs of skin retraction, or the rare but more specific skin bulge any sinus tract sign^(7,8)^.

When fat necrosis is suspected, the clinical correlation is important, due to the variable presentation of the lesions, mainly in ultrasound although also in mammography, as shown in the present case. In our patient, the need for such correlation became apparent because of the presence of comorbidities, which broadened the range of differential diagnoses.

## References

[1] Hogge JP, Robinson RE, Magnant CM (1995). The mammographics spectrum of fat necrosis of the
breast. Radiographics.

[2] Taboada JL, Stephens TW, Krishnamurthy S (2009). The many faces of fat necrosis in the breast. AJR Am J Roentgenol.

[3] Upadhyaya VS, Uppoor R, Shetty L (2013). Mammographic and sonographic features of fat necrosis of the
breast. Indian J Radiol Imaging.

[4] Dilaveri CA, Mac Bride MB, Sandhu NP (2012). Breast manifestations of systemic diseases. Int J Womens Health.

[5] Cao MM, Hoyt AC, Bassett LW (2011). Mammographic signs of systemic disease. Radiographics.

[6] Wani AM, Mohd Hussain WM, Fatani MI (2009). Lupus mastitis - peculiar radiological and pathological
features. Indian J Radiol Imaging.

[7] Khanna R, Prasanna GV, Gupta P (2002). Mammary tuberculosis: report on 52 cases. Postgrad Med J.

[8] Makanjuola D, Murshid K, Al Sulaimani S (1996). Mammographic features of breast tuberculosis: the skin bulge and
sinus tract sign. Clin Radiol.

